# Prevalence and predictors of potentially inappropriate prescribing in middle-aged adults: a repeated cross-sectional study

**DOI:** 10.3399/BJGP.2020.1048

**Published:** 2021-05-18

**Authors:** Amandeep Khatter, Frank Moriarty, Mark Ashworth, Stevo Durbaba, Patrick Redmond

**Affiliations:** School of Population Health and Environmental Sciences, King’s College London, London, UK.; School of Pharmacy and Biomolecular Sciences;; School of Population Health and Environmental Sciences, King’s College London, London, UK.; School of Population Health and Environmental Sciences, King’s College London, London, UK.; Department of General Practice, Royal College of Surgeons in Ireland, Dublin, Ireland.

**Keywords:** anti-inflammatory agents, drug-related side effects and adverse reactions, general practice, inappropriate prescribing, middle aged, multimorbidity, non-steroidal, polypharmacy

## Abstract

**Background:**

Potentially inappropriate prescribing (PIP) is common in older adults and known to be associated with polypharmacy and multimorbidity. Less is known about the prevalence and causes of PIP in middle-aged adults.

**Aim:**

To determine the prevalence and predictors of PIP in middle-aged adults.

**Design and setting:**

A repeated cross-sectional study was conducted using primary care data in London.

**Method:**

PIP was defined using the PRescribing Optimally in Middle-aged People’s Treatments (PROMPT) criteria. Prescribing and demographic data were extracted from Lambeth DataNet (LDN), a pseudonymised database of all patients registered at general practices in Lambeth, for those aged 45–64 years prescribed ≥1 medicines in each year from 2014–2019 (*n* = 46 633–52 582). Prevalence and trends over 6 years were investigated, including the association of PIP with polypharmacy, multimorbidity, deprivation, sex, and age.

**Results:**

The prevalence of PIP decreased from 20% in 2014 to 18% in 2019. The most prevalent PROMPT criteria in 2019 were the use of ≥2 drugs from the same pharmacological class (7.6%), use of non-steroidal anti-inflammatory drugs for >3 months (7.1%) and use of proton pump inhibitors above recommended maintenance dosages for >8 weeks (3.1%). Over the study period, the prevalence of multimorbidity increased (47–52%) and polypharmacy remained stable (27%). Polypharmacy, multimorbidity, deprivation, and age were independently associated with PIP. Sex was the only variable not associated with PIP.

**Conclusion:**

Almost one-fifth of middle-aged adults prescribed medicines are exposed to PIP, as defined by the PROMPT criteria. This is likely to be linked with exposure to avoidable adverse drug events. The PROMPT criteria may provide a useful aid in interventions to optimise prescribing.

## INTRODUCTION

Potentially inappropriate prescribing (PIP) involves using medicines that may cause more harm than benefit, are not cost-effective, or are not clinically indicated.^1^ Increased hospitalisation, adverse drug events (ADEs), and emergency department visits have been reported as detrimental effects of PIP.^2^ PIP is costly; costs are incurred through the use of unnecessary medicines or additional healthcare utilisation.^3^ High-risk prescribing (prescriptions most likely to cause ADEs) also varies between prescribers, with differences reported between general practices and between individual GPs.^4^

PIP in older adults (aged ≥65) has been extensively studied, with possibly 20% of prescriptions given to older adults categorised as potentially inappropriate.^5^ Polypharmacy (commonly defined as taking ≥4 medicines daily) and multimorbidity (commonly defined as having >1 long-term condition [LTC]) are strongly associated with PIP in older adults.^6^^–^^10^ However, multimorbidity is not confined to older adults, with 30% of middle-aged (45–64 years) adults also having multimorbidity.^11^ Indeed, in absolute terms, there are more people with multimorbidity aged under 65 than over.^11^ Similarly, Cooper and others^12^ found that, in 2012, 20% of middle-aged adults in Northern Ireland experienced polypharmacy.

Despite the prevalence of multimorbidity and polypharmacy, there has been limited research on the prevalence of PIP within middle-aged adults. The PRescribing Optimally in Middle-aged People’s Treatments (PROMPT) criteria were developed to estimate the prevalence of PIP in this age group (see Supplementary Table S1 for details).^13^ PROMPT specifies 22 criteria for PIP, covering a broad range of drug classes, which includes a broad range of human physiological systems, as well as duplications in drug classes, for example, strong opioids should not be prescribed without the concurrent prescription of a laxative.^13^

Previous studies have suggested that the prevalence of PIP using PROMPT ranges from 21.1–42.9%, with increased rates associated with polypharmacy, age, multimorbidity, and female sex.^12^^,^^14^^–^^16^ However, these studies were cross-sectional and drawn from selected population groups. There have been no longitudinal studies completed using PROMPT in the UK.

The aim of this study was to measure the prevalence and potential predictors of PIP defined using the PROMPT criteria in middle-aged adults.

**Table:** How this fits in

Potentially inappropriate prescribing (PIP) can lead to adverse drug events, as well as increased hospitalisation and healthcare costs. There is limited research on the prevalence and predictors of PIP in middle-aged adults. This study found that PIP is not confined to older adults, and is common in middle-aged adults too. It is also more likely in older, socioeconomically deprived patients, as well as those with polypharmacy and multimorbidity. These findings will help GPs to identify patients at increased risk of PIP in middle age.

## METHOD

### Study design and setting

A repeated cross-sectional study was conducted using general practice-derived data from Lambeth DataNet (LDN) and reported as per the STrengthening the Reporting of OBservational studies in Epidemiology (STROBE) checklist.^17^ LDN contains the pseudonymised patient records of all 41 general practices in Lambeth Clinical Commissioning Group (1 185 335 patients aged ≥18, excluding the 3.8% of patients who have opted out of data sharing) and has been extensively used in database research.^18^^,^^19^ The data used were confined to patients prescribed ≥1 medicine and aged 45–64 years in each of the years 2014–2019 inclusive. Demographically, 65% of Lambeth’s population are socioeconomically deprived (deprivation score in the bottom two quintiles), 52% are female, and over half belong to a white ethnic group.

### Outcomes

The primary outcome was the prevalence and types of PIP as described by the PROMPT criteria. PIP was explored as both a dichotomous and count variable. The secondary outcome was the association of the variables age, sex, multimorbidity, polypharmacy, and deprivation with both the presence of PIP (binary variable) and with a count of PIP over the 6 years.

### Covariates

The models were adjusted for important covariates identified a priori from literature. Age was explored in the regression models as a categorical variable. Multimorbidity was defined as those with ≥2 of 32 LTCs (see Supplementary Box S1 for details). This definition was derived from Cassell and others’^20^ codes and modified following local consultation (hence inclusion of sickle cell disease and lupus). For Quality and Outcomes Framework (QOF) conditions, the study accepted QOF definitions of Read and Systematized Nomenclature of Medicine Clinical Terms (SNOMED-CT). For the conditions that rely on medication code (for example, chronic pain) Egton Medical Information Systems codes were used (data available on reasonable request). The definition of polypharmacy, used as a count variable rather than implying appropriateness, was those prescribed ≥4 repeat medicines in a year.^6^ Sex was included as a binary variable. Deprivation was analysed as the locally determined Index of Multiple Deprivation quintile, which is assigned based on lower layer super output areas of residence of each patient. The distribution of individuals in the study population was analysed by each of these variables and PIP.

### Analysis

Descriptive statistics were used to describe the prevalence of PIP in the study population. The percentages calculated for each of the PROMPT criteria represented the study population prevalence.

Unadjusted and adjusted logistic regression was performed to assess the association of PIP with study year (using 2014 as the reference year), age group, sex, deprivation, multimorbidity, and polypharmacy. A cluster variable incorporating the patient ID with the code for their general practice was created to allow for intraclass correlation and was included in the regression model. Odds ratios with 95% confidence intervals (CI) are presented.

Negative binomial regression was used to quantify the change in the rate of PIP associated with the included covariates. Incidence rate ratios (IRR) with 95% CI are presented. A negative binomial model was used over a Poisson model owing to overdispersion of PIP rates.

Data were checked for non-random missingness with no imputation required. Regression diagnostics were run to ensure goodness of fit. All statistical analyses were carried out using Stata 14.

## RESULTS

### Descriptive

The number of individuals included in this study ranged from 46 633 in 2014 to 52 582 in 2019. Table 1 shows the descriptive statistics for the population. The study population was positively skewed towards the younger age categories, with 32% aged 45–49 and 17% aged 60–64. Similarly, there was a decreasing proportion of individuals living in the most deprived decile (22%) compared with the least deprived decile (18%).

**Table 1.** Study population characteristics

**Descriptive**	**2014**	**2015**	**2016**	**2017**	**2018**	**2019**
**Sex, *n* (%)**						
Female	24 676 (52.92)	25 352 (52.77)	25 937 (52.61)	26 522 (52.61)	27 134 (52.75)	27 739 (52.75)
Male	21 957 (47.08)	22 694 (47.23)	23 363 (47.39)	23 892 (47.39)	24 307 (47.25)	24 843 (47.25)

**Age group, n (%)**						
45–49	14 804 (31.75)	14 682 (30.56)	14 386 (29.18)	14 247 (28.26)	12 877 (26.98)	13 718 (26.09)
50–54	13 661 (29.29)	14 059 (29.26)	14 523 (29.47)	14 639 (29.04)	14 729 (28.63)	14 706 (27.97)
55–59	10 381 (22.26)	11 090 (23.08)	11 571 (23.47)	12 227 (24.25)	12 857 (24.99)	13 532 (25.74)
60–64	7787 (16.70)	8215 (17.10)	8820 (17.89)	9301 (18.45)	9978 (19.40)	10 626 (20.21)

**Multimorbidity (≥2 LTCs)**	21 775 (46.69)	22 905 (47.67)	24 158 (49.00)	25 157 (49.90)	26 263 (51.05)	27 140 (51.61)

**Polypharmacy (≥4 repeat medicines)**	12 651 (27.13)	13 078 (27.22)	13 547 (27.48)	13 912 (27.60)	14 201 (27.61)	14 588 (27.74)

**Deprivation quintile^a^**						
1 (least deprived)	10 035 (21.61)	10 337 (21.61)	10 620 (21.61)	10 766 (21.41)	10 805 (21.10)	10 980 (20.97)
2	9607 (20.68)	9938 (20.77)	10 441 (21.26)	10 349 (20.61)	10 943 (21.37)	11 112 (21.22)
3	9445 (20.34)	9666 (20.20)	9688 (19.73)	10 220 (20.36)	10 139 (19.80)	10 278 (19.63)
4	8922 (19.21)	9188 (19.21)	9547 (19.44)	9715 (19.35)	10 074 (19.67)	10 459 (19.98)
5 (most deprived)	8436 (18.16)	8712 (18.21)	8810 (17.94)	9153 (18.23)	9255 (18.07)	9529 (18.20)

**Total PIP prevalence**	9324 (19.99)	9644 (20.07)	9752 (19.78)	9716 (19.27)	9430 (18.33)	9582 (18.22)

a *There was missing data (0.4%) for the deprivation variable.*

**Figure 1.**
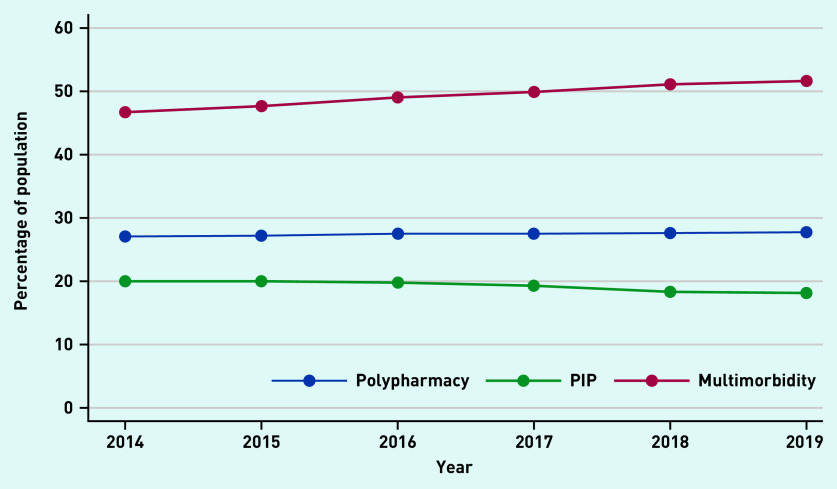
*Prevalence of polypharmacy, multimorbidity, and PIP over the study period.* *PIP = potentially inappropriate prescribing.*

Figure 1 shows the percentage of the study population with PIP, polypharmacy, and multimorbidity over time. There was an increase in the proportion of individuals with ≥2 LTCs, which rose from 46.7–51.6% (see Supplementary Figure S1 for details). There was a small increase in those prescribed ≥4 repeat medicines; however, this generally remained consistent at 27%. The prevalence of PIP decreased, but the proportion still remained high (>18%). The absolute number of people with one PIP increased over the 6 years (*n* = 5802–6391, ∼12%) Between 4–5% of the population had two and 2–3% had ≥3 PIPs (see Supplementary Table S2 for details).

### Primary outcome

**Figure 2.**
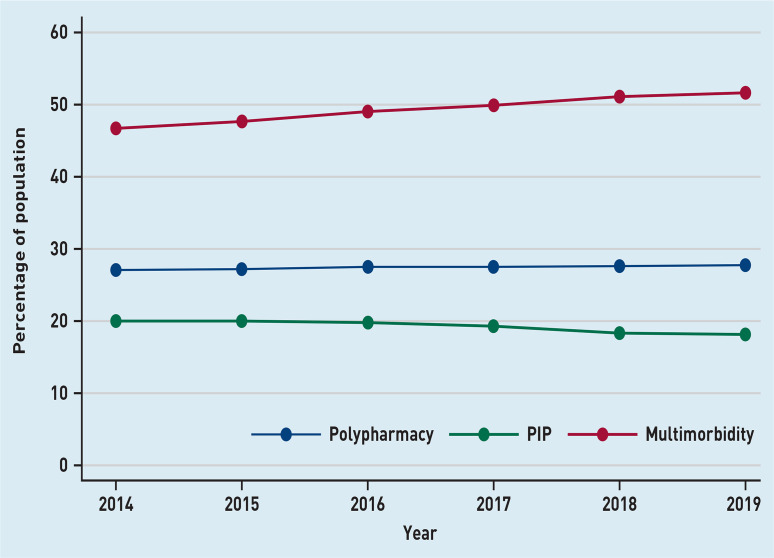
*Prevalence of the eight most common PROMPT criteria.* *GI = gastrointestinal. NSAID = non-steroidal anti-inflammatory drug. PPI = proton pump inhibitor. PROMPT = PRescribing Optimally in Middle-aged People’s Treatments. SSRI = selective serotonin reuptake inhibitor.*

Percentage prevalence estimates were calculated for each PROMPT criterion (see Supplementary Table S2 for details). Of the 22 criteria, 14 had a percentage prevalence ≤0.5% over the 6 years. The eight most commonly occurring examples of PIP are shown in Figure 2.

**Figure 3.**
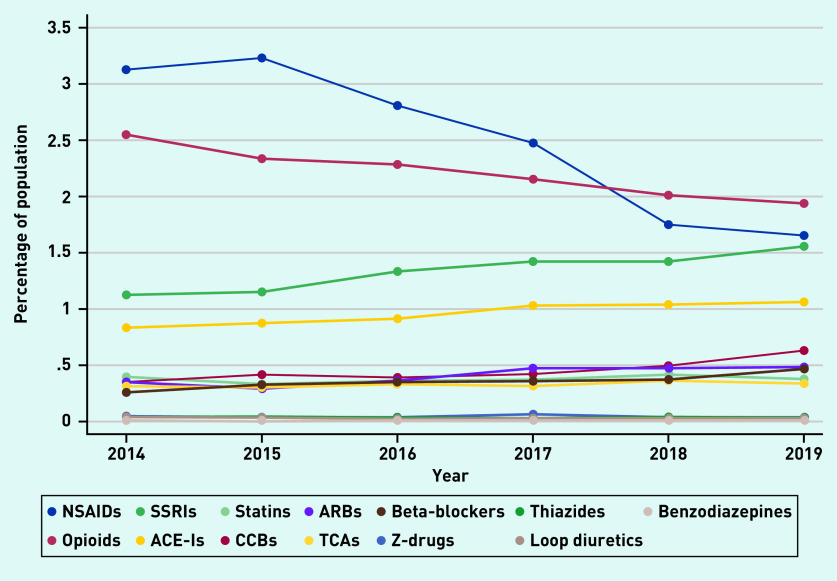
*Prevalence of duplication of drug classes.* *ACE-I = angiotensin-converting enzyme inhibitor. ARB = angiotensin receptor blocker. CCB = calcium channel blocker. NSAID = non-steroidal anti-inflammatory drug. SSRI = selective serotonin reuptake inhibitor. TCA = tricyclic antidepressant.*

In 2014, the most prevalent PROMPT criterion was the use of non-steroidal anti-inflammatory drugs (NSAIDs) for >3 months (*n* = 4458, 9.6%). The prevalence decreased over the 6 years to 7.1%. By 2019, the concurrent use of ≥2 drugs from the same pharmacological class was the most common criterion (*n* = 4003, 7.6%); however, even this represented a decrease from 2014 (8.4%). The repeated prescription of ≥2 NSAIDs contributed the most to this PROMPT criterion, followed by opioids and selective serotonin reuptake inhibitors (SSRIs) (Figure 3). The use of proton pump inhibitors (PPIs) above the recommended maintenance dosage for >8 weeks was the only PROMPT criterion to increase over the study period (from 2.6% to 3.1%).

### Analysis

In the adjusted logistic regression model, a trend of reducing odds of PIP was observed across the study years (Table 2). Polypharmacy was the most strongly associated variable (adjusted odds ratio [AOR] = 4.85, 95% CI = 4.69 to 5.02), followed by multimorbidity (AOR = 2.61, 95% CI = 2.52 to 2.71). Further analysis of the multimorbidity covariate identified that coronary heart disease (AOR = 5.65, 95% CI = 5.12 to 6.24), peripheral arterial disease (AOR = 2.44, 95% CI = 1.99 to 3.00), and chronic pain (AOR = 2.35, 95% CI = 2.25 to 2.44) were the most significantly associated with PIP (see Supplementary Table S3 for details). Those aged 60–64 were 20% more likely (AOR = 1.19, 95% CI = 1.14 to 1.25) to have PIP compared with those aged 45–49. Living in the most deprived areas increased the odds of PIP (AOR = 1.17, 95% CI = 1.11 to 1.23) compared with the least deprived areas. There was no statistically significant association between PIP and female sex (AOR = 1.01, 95% CI = 0.97 to 1.04, *P* = 0.67).

**Table 2.** Logistic and negative binomial regression analyses for PIP

**Covariate**	**Adjusted logistic regression**		**Negative binomial regression**	
	
	**OR (95% CI)**	***P*-value**	**IRR (95% CI)**	***P*-value**
**Year of study**				
2014 (reference)	1	—	1	—
2015	0.99 (0.97 to 1.02)	0.600	0.99 (0.97 to 1.01)	0.256
2016	0.95 (0.93 to 0.98)	0.001	0.95 (0.93 to 0.97)	<0.001
2017	0.90 (0.88 to 0.93)	<0.001	0.91 (0.89 to 0.93)	<0.001
2018	0.83 (0.80 to 0.86)	<0.001	0.83 (0.81 to 0.85)	<0.001
2019	0.81 (0.79 to 0.84)	<0.001	0.82 (0.80 to 0.84)	<0.001

**Polypharmacy**	4.85 (4.69 to 5.02)	<0.001	3.91 (3.80 to 4.03)	<0.001

**Multimorbidity**	2.61 (2.52 to 2.71)	<0.001	2.47 (2.38 to 2.55)	<0.001

**Female sex**	1.01 (0.97 to 1.04)	0.651	1.03 (1.01 to 1.06)	0.016

**Age group**				
45–49 (reference)	1	—	1	—
50–54	1.05 (1.01 to 1.08)	0.017	1.03 (0.99 to 1.06)	0.111
55–59	1.10 (1.05 to 1.14)	<0.001	1.06 (1.02 to 1.09)	0.002
60–64	1.19 (1.14 to 1.25)	<0.001	1.11 (1.07 to 1.15)	<0.001

**Deprivation quintile**				
5 (reference, least deprived)	1	—	1	—
4	1.09 (1.03 to 1.15)	0.002	1.09 (1.04 to 1.14)	<0.001
3	1.14 (1.08 to 1.20)	<0.001	1.12 (1.07 to 1.17)	<0.001
2	1.18 (1.12 to 1.25)	<0.001	1.17 (1.12 to 1.23)	<0.001
1 (most deprived)	1.17 (1.11 to 1.23)	<0.001	1.16 (1.11 to 1.21)	<0.001

*CI = confidence interval. OR = odds ratio. IRR = incidence rate ratio.*

Within the negative binomial model, associations of all variables with the rate of PIP were of a similar magnitude and direction, except for female sex. Here, the parameter estimates were analogous to the logistic model, but reached statistical significance in the negative binomial regression (IRR = 1.03, 95% CI = 1.01 to 1.06, *P* = 0.02).

The intraclass correlations at the patient and practice level were 0.59 and 0.02 respectively (Table 2), indicating greater correlation between observations from the same patient than among observations from the same general practice.

## DISCUSSION

### Summary

At least 18% of the study population were prescribed a potentially inappropriate prescription in each year. Across the 6 years of the study, the prevalence of multimorbidity (47–52%) and polypharmacy (27%) was high. In 2019, the three most common PIPs were the use of ≥2 drugs concurrently from the same pharmacological class, the use of NSAIDs for >3 months, and the use of PPIs above the recommended maintenance dosage for >8 weeks. Having polypharmacy (AOR = 4.85, IRR = 3.91) multimorbidity (AOR = 2.61, IRR = 2.47), living in a more deprived area, and being older significantly increased the odds and incidence rates of PIP.

### Strengths and limitations

This is the first study to report trends in PIP in middle-aged adults over time. It is also the first to adjust for the clustering of patients and general practices within the regression models and to explore deprivation as an explanatory variable. The use of patient record data (rather than purely prescribing or dispensing datasets) allowed for the examination of additional predictors such as deprivation and multimorbidity. However, patient record data do not include over-the-counter medicines use and may not directly relate to actual dispensing or patient adherence.

Another potential limitation involved the extraction parameter assumptions. It was assumed that the number of prescriptions given equated to a time period for a particular medicine. Therefore, the calculated prevalence for some of the criteria may be underestimated, as some patients may have been given long-duration prescriptions. Additionally, it was assumed that patients prescribed ≥2 medicines from the same drug class multiple times (≥3 prescriptions of each medicine within 6 months) was synonymous with concomitant use. It should be noted, however, that this approach is consistent with previous studies.^12^

The PROMPT criteria are limited by their explicit nature, as they do not take into account patients’ or prescribers’ preferences. This implies that some prescriptions categorised as potentially inappropriate may be justified and appropriate when taking individual patient circumstances into account. For example, there may be clinical rationale to multiple alternative analgesics in chronic pain conditions, but this would be identified as PIP under the duplicate drug class criterion.^13^ However, PROMPT is the only tool developed for use specifically in middle-aged adults, and many of the individual criteria overlap with extensively researched tools such as the Beers Criteria.^21^

The study population was also limited to those prescribed ≥1 medicine; therefore, the multimorbidity prevalence would be expected to be higher than the adult population average. Finally, the validity of the findings is supported by the prevalence of polypharmacy (27%) being consistent with existing studies.^14^^,^^22^

### Comparison with existing literature

The literature analysing PIP in middle-aged adults is very limited. Only four previous studies measured the prevalence of PIP using the PROMPT criteria.^12^^,^^14^^–^^16^ This study reported a lower prevalence of PIP (18%) compared with previous studies (ranging from 21.1–42.9%). Harasani and others^15^ looked at the dispensing data of a selection of community pharmacies in Albania, and Moriarty and others^14^ analysed participants from a means-tested scheme in Ireland, with the representativeness of these studies limited owing to their selected population.

This study found that the use of ≥2 drugs concurrently from the same pharmacological class was one of the most prevalent PIPs. Previous studies reported that NSAIDs and opioids are commonly implicated in this criterion, similar to this study.^12^^,^^14^ In contrast to previous studies, the prescribing of duplicates of benzodiazepines was found to be negligible. Furthermore, the use of NSAIDs for >3 months and the use of PPIs above the recommended maintenance dosage for >8 weeks commonly occurred, as in existing studies.^12^^,^^14^^,^^15^

Research has consistently shown a positive association between PIP and polypharmacy, in both middle-aged and older adults.^12^^,^^23^ The relationship between multimorbidity and PIP in middle-aged adults is less well understood; however, when it has been included in previous studies, it also shows a positive association with PIP.^14^^,^^24^ Both female sex and increasing age are inconsistently associated with PIP in older adults, but studies in middle-aged adults found a positive association.^12^^,^^14^^,^^23^ The observed increased odds of PIP with greater deprivation confirms the arguments raised by Cooper and others^12^ in their comparative study of two populations differing in their deprivation levels.

### Implications for research and practice

It is known that ADEs are associated with PIP in middle-aged adults.^16^ This study provides evidence that the prevalence of PIP in middle-aged adults is high. Therefore, intervening to optimise prescribing in this age group may reduce these ADEs.

National Institute for Health and Care Excellence guidelines on medicines optimisation advise using screening tools to monitor PIP in older adults and those with polypharmacy.^25^ The results of this study underline that PIP is not confined to older adults, and so there should also be a specific focus in the guidelines on the role of tools such as PROMPT in middle-aged adults.

Currently, prescribing optimisation interventions are primarily focused on older adults, and aim to increase awareness of PIP and improve prescribing appropriateness.^26^^,^^27^ Given the common nature of PIP in middle-aged adults, future research should investigate the benefit of primary care-based interventions to improve prescribing in this group also. For example, clinical decision support (CDS) tools could incorporate the PROMPT criteria. However, CDS tools are known to have limitations, notably that prescribers can override and ignore them.^28^^,^^29^

PIP of NSAIDs and PPIs relate to long-term use, which could be addressed through frequent medication reviews. A meta-analysis of medicine reviews has found that they can reduce PIP-related ADEs.^30^ Given the coexistence of PIP, multimorbidity, polypharmacy, and deprivation, targeting the delivery of these interventions may improve their effectiveness. This may be supported by future research further delineating disease patterns most at risk of PIP. However, reviews can be time consuming and specialised, requiring careful consideration of risk/benefit profiles. Increasingly, the role of practice-based pharmacists in performing reviews is recognised,^31^ allowing more thorough reviews and increased patient participation.^32^
